# Referred Shoulder Pain Due to the Positioning of the Ventriculoperitoneal Shunt: A Case Report and Literature Review of an Unusual Complication

**DOI:** 10.7759/cureus.63819

**Published:** 2024-07-04

**Authors:** Qais A Samara, Sultan Jarrar, Suleiman S Daoud, Yousef M Odeibat, Amer A Alomari

**Affiliations:** 1 Neurosurgery, Department of Special Surgery, Faculty of Medicine, Al-Balqa Applied University, Al-Salt, JOR; 2 Neurosurgery, Faculty of Medicine, Jordan University of Science and Technology, Irbid, JOR; 3 Neurosurgery, Neuron Clinics, Amman, JOR; 4 Neurosurgery, Department of Special Surgery, Faculty of Medicine, Mutah University, Al-Karak, JOR

**Keywords:** phrenic nerve, shoulder pain, diaphragm, ventriculoperitoneal (vp) shunt, kehr's sign, shoulder tip pain, referred pain, ventriculoperitoneal shunt complications, hydrocephalous

## Abstract

We present the case of an 18-year-old male with a ventriculoperitoneal (VP) shunt for hydrocephalus who experienced right shoulder pain. The patient was thoroughly investigated for gastrointestinal disease, including abdominal ultrasound and upper endoscopy, which revealed no abnormalities that could explain his symptoms. X-ray imaging subsequently revealed that the shunt’s distal peritoneal tubing was positioned in a supra-hepatic subdiaphragmatic location. Surgical shortening and repositioning of the peritoneal tubing successfully alleviated the patient's shoulder pain.

A review of the literature uncovered four articles, comprising a total of six patients, who exhibited similar symptoms of shoulder pain linked to their VP shunts. Given the rarity of this complication, it can be easily overlooked or misdiagnosed. It is crucial for physicians to consider this possibility when evaluating patients with VP shunts who present with shoulder pain to ensure prompt and effective treatment.

## Introduction

Hydrocephalus is a neurological condition defined by the active distension of the brain’s ventricular system due to inadequate passage of cerebrospinal fluid (CSF) from its production site to its absorption into the systemic circulation. This disruption results in CSF accumulation, typically due to obstructions in the CSF pathway [1]. This accumulation results in elevated intracranial pressure, which, if not promptly managed, can lead to considerable neuronal and axonal damage, particularly in the periventricular white matter, ultimately impairing cognitive functions and motor skills [2].

Ventriculoperitoneal (VP) shunts are commonly used to manage hydrocephalus by diverting excess CSF from the brain to the peritoneal cavity, thereby reducing intracranial pressure and preventing adverse effects [3]. Despite the effectiveness of VP shunts, complications can include infection, shunt obstruction, abdominal pseudocysts, hematomas, ascites, CSF leaks, and shunt migration [4].

This case report highlights an unusual presentation of diaphragmatic irritation leading to referred shoulder pain in a patient with a VP shunt. Typically, referred shoulder pain is evaluated by general surgeons and gastroenterologists for common abdominal causes. However, in patients with VP shunts, neurosurgeons should consider the possibility of diaphragmatic irritation. Prompt recognition and appropriate surgical intervention, such as revision and shortening of the distal peritoneal tubing, can effectively alleviate the pain. This case highlights the need to consider diaphragmatic irritation as a potential cause of shoulder pain in patients with VP shunts. It emphasizes the importance of maintaining a high index of suspicion and ensuring prompt diagnosis for optimal treatment outcomes.

## Case presentation

**Figure 1:**
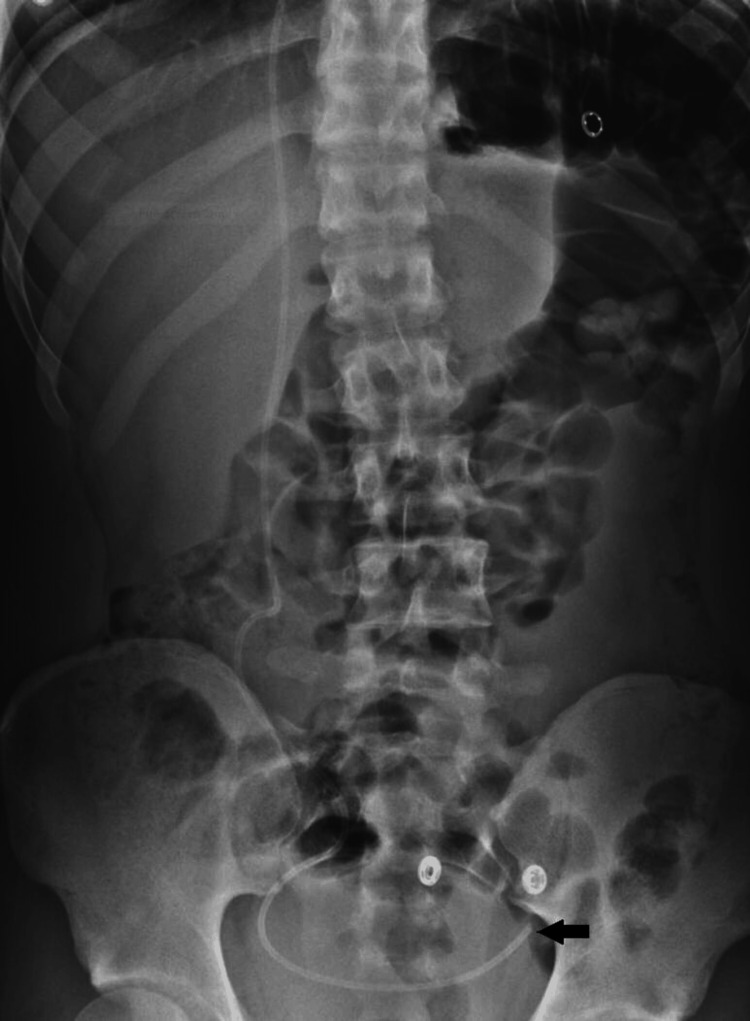
The anteroposterior abdomen X-ray demonstrates that the shunt's peritoneal tubing is satisfactorily positioned within the pelvic peritoneal space (arrow).

An 18-year-old male with hydrocephalus, who had a right parietal VP shunt inserted at six months of age presented to our attention at the emergency department 15 days after revision surgery of the shunt's distal peritoneal tubing, which had been inserted through a right periumbilical incision. He reported a one-week history of headaches, nausea, vomiting, and diplopia. On examination, bilateral abducent palsy and a noncompressible valve were noted. A brain computed tomography (CT) scan demonstrated dilated ventricles, and a shunt tap revealed elevated CSF pressure and nonfunctioning distal tubing. Surgical exploration of the shunt system revealed that the distal tubing was disconnected within the vicinity of the valve and was obstructed. Following flushing to clear the obstruction, the tubing was reconnected to the valve, confirmed to be functioning, and reinserted through the same right periumbilical incision. A postoperative shunt survey revealed that the peritoneal tubing was in a satisfactory position within the pelvic peritoneal space (Figure 1). The patient recovered smoothly and was discharged on postoperative day three.

**Figure 2:**
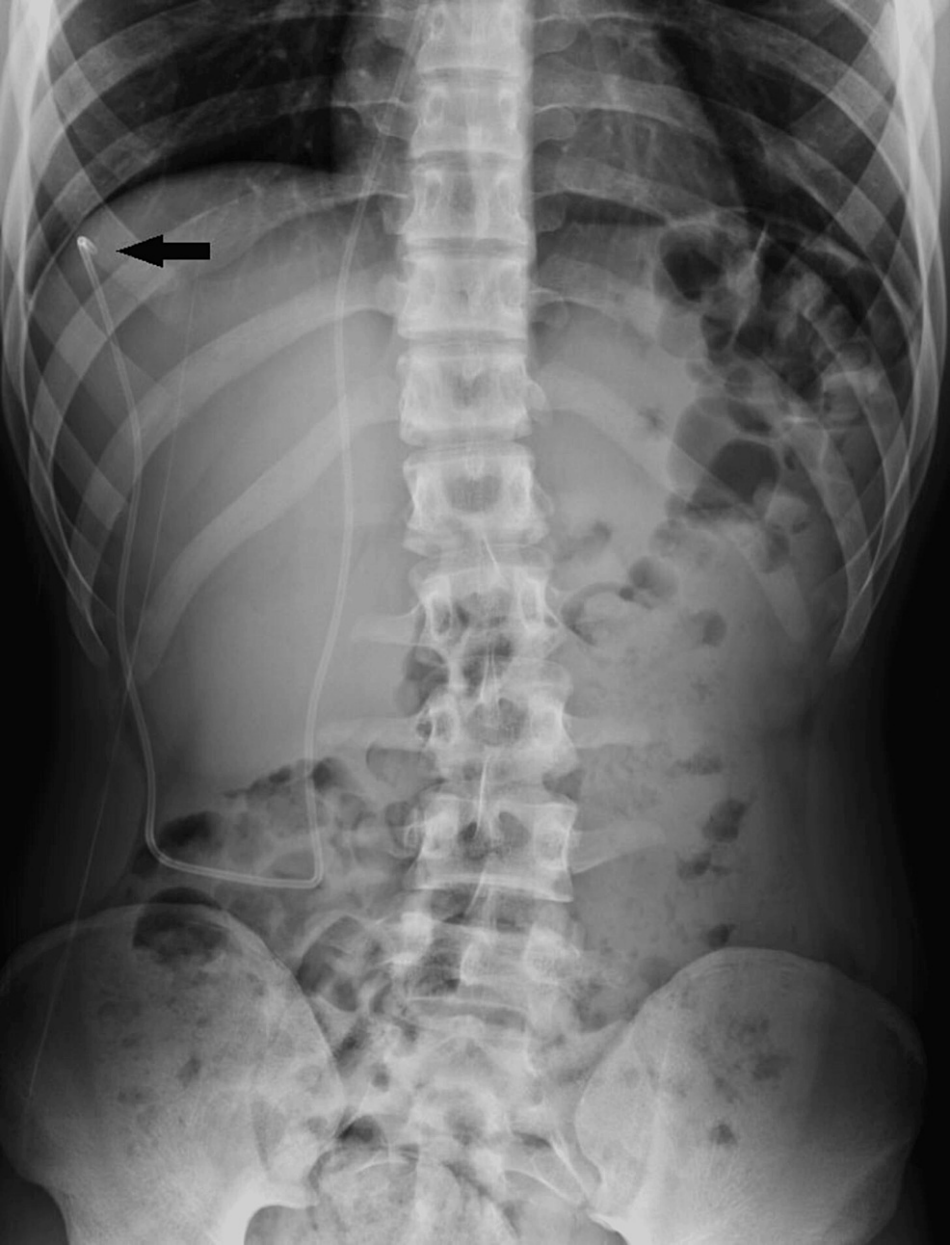
The anteroposterior abdomen X-ray shows an upward migration of the peritoneal tubing to the right paracolic gutter (arrow).

Two days after discharge, the patient returned to the emergency department with complaints of abdominal pain and constipation. On physical examination, the rectum was full of stools. The shunt survey indicated that the tip of the distal tubing had migrated upward into the right paracolic gutter, as seen in Figure 2. The patient was discharged home with laxatives.

Nineteen days later, the patient returned to the emergency department with a severe headache that had begun one day earlier. A brain CT scan revealed slit-like ventricles with a small, mixed-density left subdural hematoma. The patient underwent a shunt valve replacement, switching to a Medtronic Strata II programmable valve set (Medtronic, Minneapolis, MN) to a performance level of two. Postoperatively, the patient recovered smoothly; however, on postoperative day two, he developed right shoulder pain, which was managed conservatively with simple analgesics. The patient was discharged on postoperative day three.

Sixteen days later, the patient continued to complain of right shoulder pain associated with epigastric pain, accompanied by nausea and vomiting. The symptoms were exacerbated by food and partially alleviated by proton pump inhibitors. The gastroenterology team was consulted, and an abdominal ultrasound reported no pathology involving the gallbladder. An upper abdomen endoscopy revealed pangastritis and a gastric biopsy showed chronic moderate-to-severe inflammation without evidence of *Helicobacter pylori* infection. The gastroenterologist prescribed esomeprazole and domperidone to manage the patient's symptoms. Additionally, a shoulder X-ray and shunt evaluation were performed, and both were reassuring.

**Figure 3:**
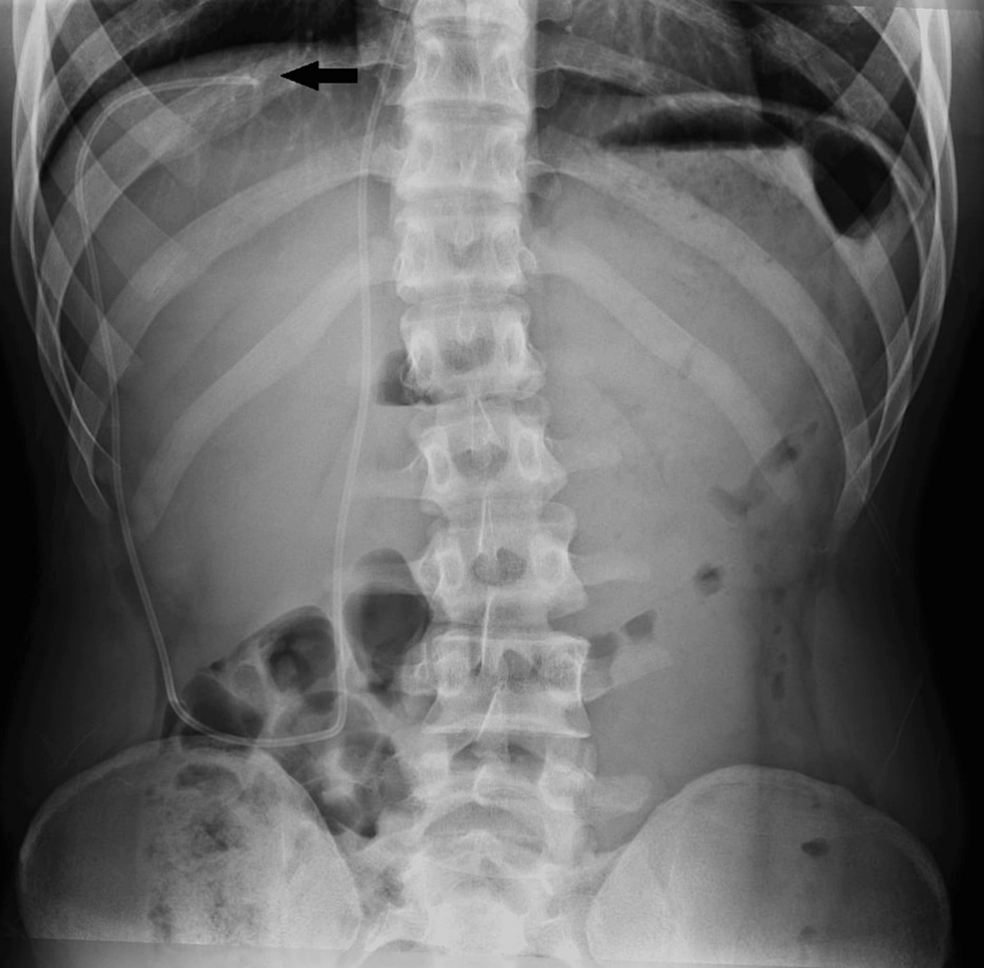
An anteroposterior abdomen X-ray shows the peritoneal tubing positioned in the subdiaphragmatic supra-hepatic recess (arrow).

After four and a half months of medical treatment, the abdominal and right shoulder pain persisted. A right shoulder MRI was unremarkable. An abdomen X-ray showed that the peritoneal tubing was positioned in the subdiaphragmatic supra-hepatic recess of the peritoneal cavity, as seen in Figure 3. It was hypothesized that the tubing’s position might be causing irritation to the diaphragm, leading to referred pain in the right shoulder. To address this issue, the patient underwent revision surgery of the distal peritoneal tubing, which involved reopening the right periumbilical wound, externalizing the distal tubing, shortening it by approximately 5 centimeters, and reinserting the tubing through the same incision but with a new, more medial peritoneal insertion site.

**Figure 4:**
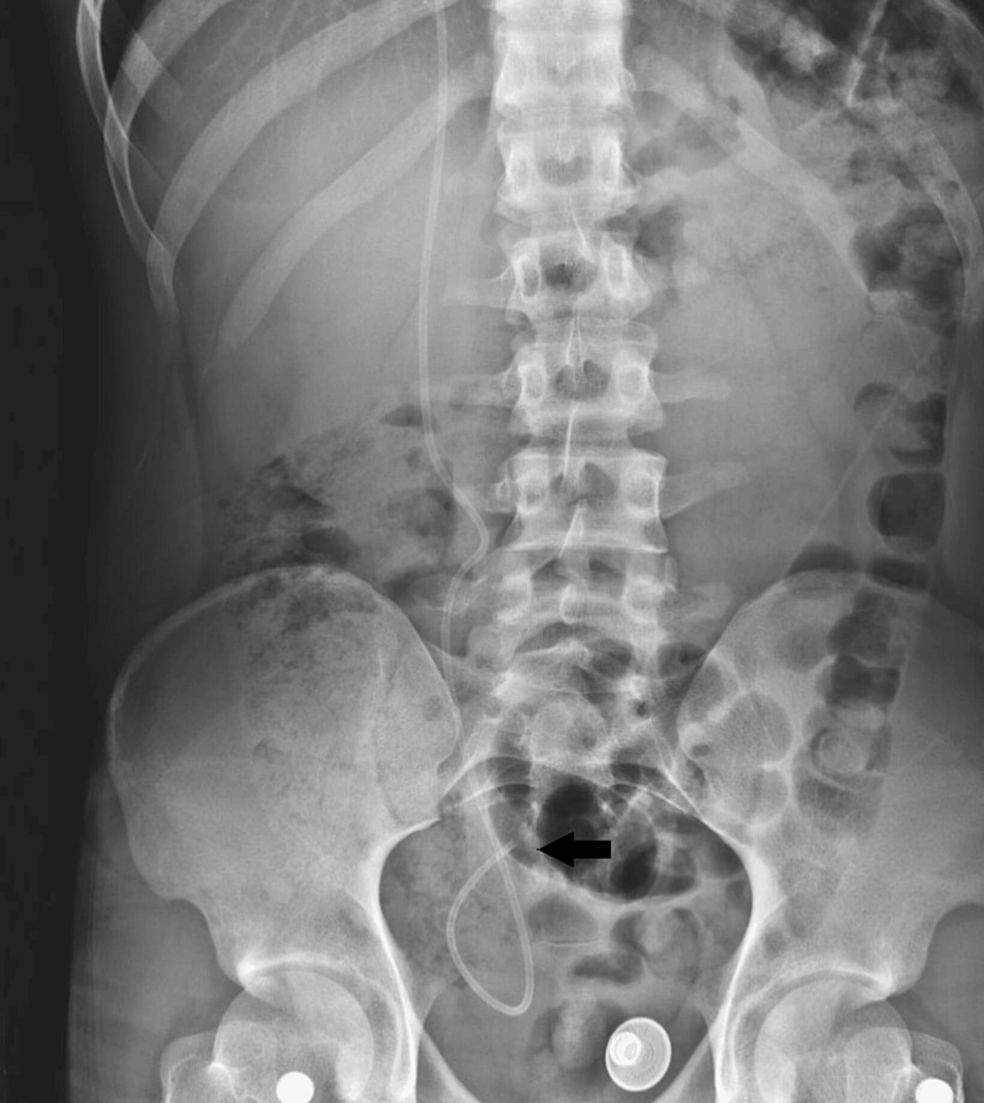
An anteroposterior abdomen X-ray shows the shunt’s peritoneal catheter satisfactorily positioned within the pelvic peritoneal cavity (arrow).

After recovering from the surgery, the patient’s abdominal and right shoulder pain were immediately relieved. A postoperative abdomen X-ray showed that the peritoneal tubing was positioned satisfactorily within the pelvic peritoneal cavity, as seen in Figure 4. At his last follow-up, four years after the surgery, the patient's right shoulder pain had not recurred.

## Discussion

The complications of VP shunts are numerous and include infection, shunt obstruction, abdominal pseudocyst, bowel perforation, overdrainage, and subdural hematoma [5]. Referred shoulder pain due to diaphragmatic irritation by the shunt’s distal peritoneal tubing is a rare complication. A literature review on PubMed yielded four articles describing six cases of referred shoulder pain in VP shunt patients, summarized in Table 1. A common observation among all cases was the subdiaphragmatic positioning of the distal peritoneal tubing, with symptoms resolving after revision surgery of the tubing.

**Table 1:** Summary of the literature review of referred shoulder pain in VP-shunted patients F: female; M: male; R: right; L: left; VA: ventriculoarterial

Reference	Case	Age (years old)	Sex	Side of shoulder pain	Duration of shoulder pain complaint	Management
Tubbs et al., 2005 [6]	1	10	F	R	4 months	Peritoneal tubing repositioned into the left lower quadrant
	2	12	F	R	4 months	Peritoneal tubing repositioned into the left lower quadrant
	3	16	M	R	4 months	Peritoneal tubing shortened and repositioned into the left lower quadrant
Lim et al., 2005 [7]	4	20	M	L	3 weeks	Peritoneal tubing repositioned
Lang et al., 2011 [8]	5	29	F	R	1 year	Peritoneal tubing repositioned into a left subcostal incision
Suzuki et al., 2020 [9]	6	9	F	R then L	1 week	Antibiotics, external drainage, VA shunt
Present case	7	18	M	R	4.5 months	Peritoneal tubing shortened and repositioned into a more medial peritoneal site

Tubbs et al. reported three pediatric cases of right shoulder pain in VP-shunted patients. Abdominal X-rays revealed the peritoneal tubing positioned between the liver and the right hemidiaphragm. Surgical repositioning of the tubing to the left lower quadrant resolved the shoulder pain in all patients [6].

Lim et al. described a 20-year-old male with hydrocephalus secondary to glioblastoma multiforme treated with bilateral VP shunts who experienced severe left shoulder pain six weeks post-surgery. The X-ray showed the right shunt’s peritoneal tubing abutting the left hemidiaphragm. Surgical repositioning of the tubing immediately resolved the pain [7].

Lang et al. reported a 29-year-old female with a VP shunt for benign intracranial hypertension who experienced intermittent right upper quadrant and shoulder pain one year post revision. Initial imaging showed the tubing in the left abdomen, and an ultrasound revealed a gallstone. Despite undergoing a laparoscopic cholecystectomy, her shoulder pain recurred. Further imaging found the shunt tube under the right diaphragm. Repositioning the tube to a left subcostal location resolved her shoulder pain immediately [8].

Suzuki et al. described a nine-year-old girl with right abdominal pain radiating to the shoulder. Abdominal X-rays revealed the tube wrapped around the liver. Initially diagnosed with peritonitis, she received antibiotics without improvement. Her pain later shifted to the left abdomen, radiating to the left shoulder. Laparoscopic repositioning and replacement with a silicone tube provided only temporary relief. Propionibacterium acnes was later identified. Eventually, she underwent an operation for external drainage and received adequate antibiotic therapy, leading to the placement of a ventriculoarterial shunt with no recurrence of her symptoms [9].

Referred pain is pain felt at a site distant from the actual source of the noxious stimulus [10]. Referred shoulder pain can originate from neurological, vascular, neurovascular, and visceral sources, including conditions affecting the lungs, heart, diaphragm, and gastrointestinal system [11]. Diaphragmatic irritation can specifically cause referred shoulder pain because it involves the phrenic nerve, which originates from the C3-C5 spinal cord segments. These spinal segments innervate both the diaphragm and the shoulder, leading to the convergence of sensory pathways. As a result, visceral pain signals from the diaphragm are perceived as somatic pain in the shoulder [12].

It is essential to recognize referred shoulder pain as a possible complication of VP shunts. Failure to be aware of this complication can lead to misdiagnosis, unnecessary investigations, and procedures, resulting in delayed treatment and a negative impact on the patient's quality of life. The take-home message is that unexplained shoulder pain in a patient with a VP shunt should prompt an investigation of the position of the distal peritoneal tubing.

## Conclusions

Referred shoulder pain resulting from diaphragmatic irritation by the distal peritoneal tubing in VP-shunted patients, though uncommon, is an important and easily managed complication. However, it requires a high index of suspicion for prompt recognition and management. Both our case and the reviewed literature demonstrate that this pain is associated with the subdiaphragmatic location of the peritoneal tubing and that repositioning the tubing provides immediate relief of symptoms. In conclusion, the investigation of the position of the distal peritoneal tubing should be initiated in the event of unexplained shoulder pain in a patient with a VP shunt.
